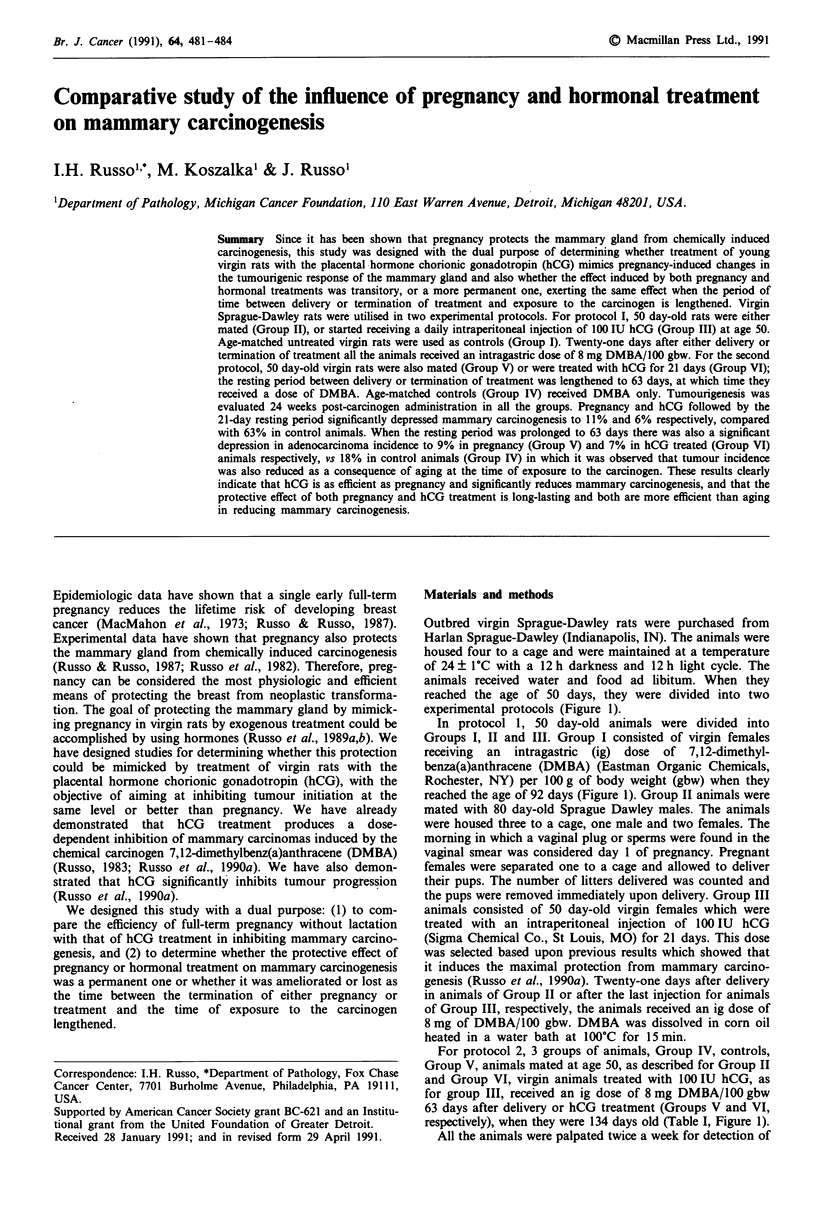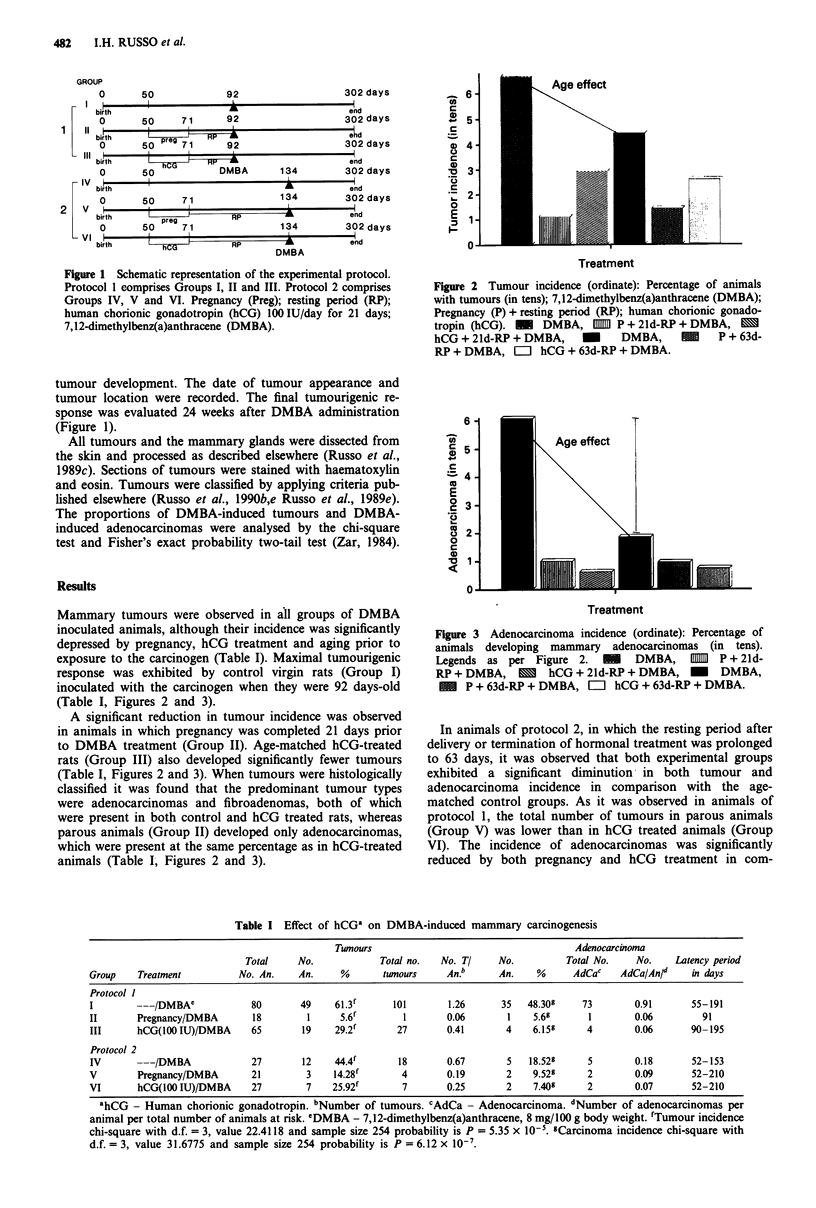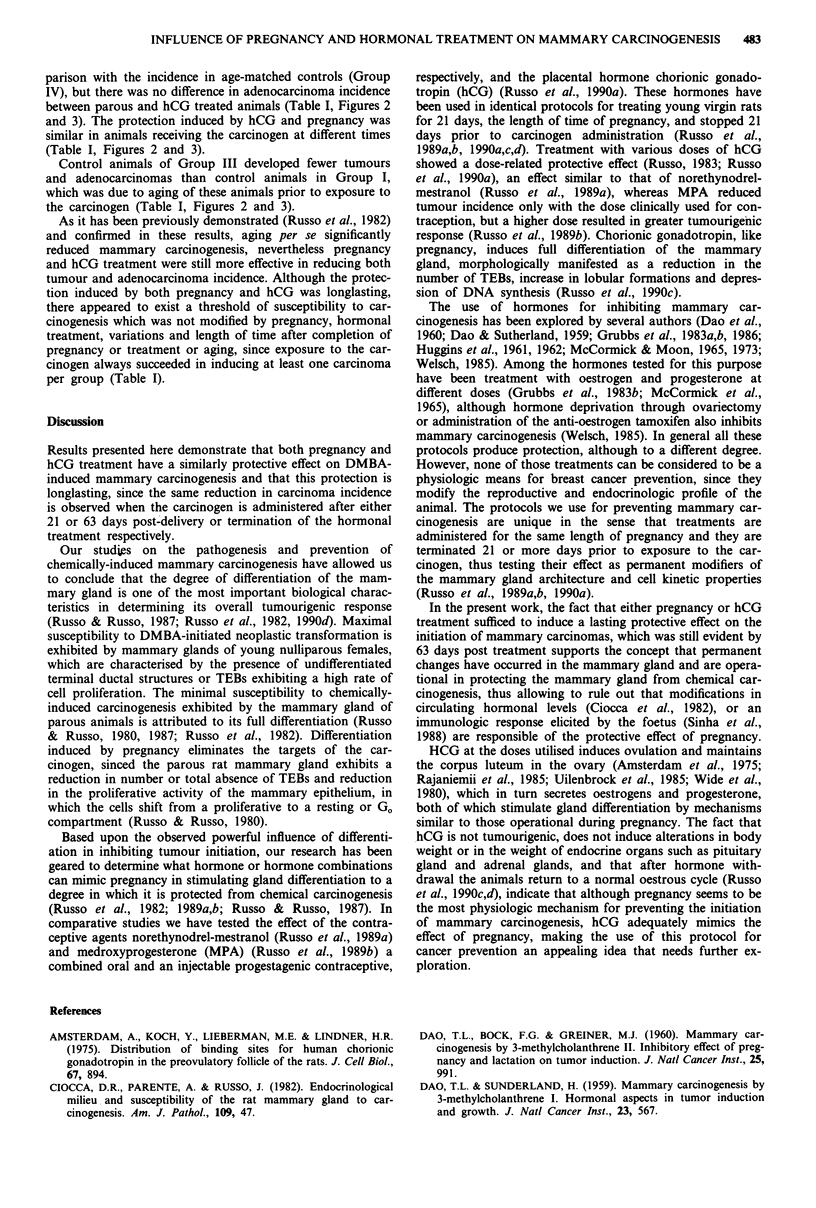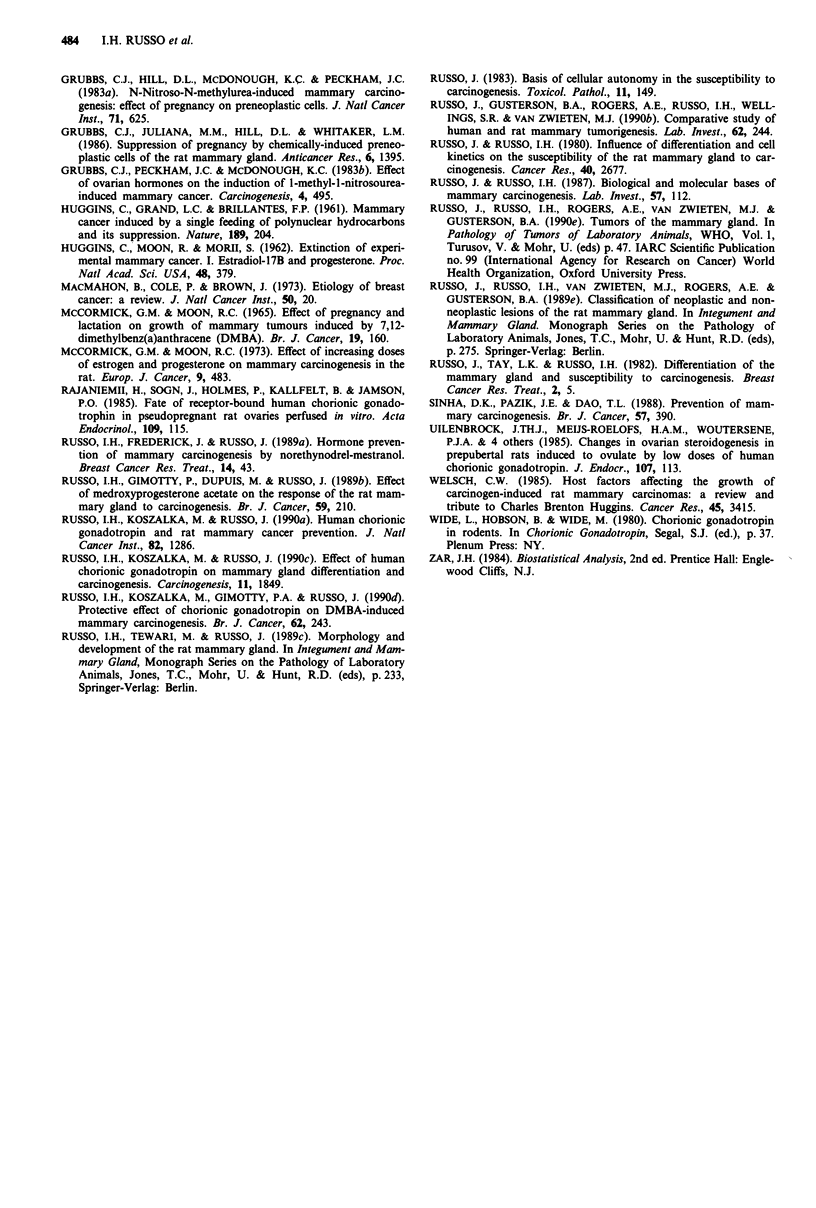# Comparative study of the influence of pregnancy and hormonal treatment on mammary carcinogenesis.

**DOI:** 10.1038/bjc.1991.335

**Published:** 1991-09

**Authors:** I. H. Russo, M. Koszalka, J. Russo

**Affiliations:** Department of Pathology, Michigan Cancer Foundation, Detroit 48201.

## Abstract

Since it has been shown that pregnancy protects the mammary gland from chemically induced carcinogenesis, this study was designed with the dual purpose of determining whether treatment of young virgin rats with the placental hormone chorionic gonadotropin (hCG) mimics pregnancy-induced changes in the tumourigenic response of the mammary gland and also whether the effect induced by both pregnancy and hormonal treatments was transitory, or a more permanent one, exerting the same effect when the period of time between delivery or termination of treatment and exposure to the carcinogen is lengthened. Virgin Sprague-Dawley rats were utilised in two experimental protocols. For protocol I, 50 day-old rats were either mated (Group II), or started receiving a daily intraperitoneal injection of 100 IU hCG (Group III) at age 50. Age-matched untreated virgin rats were used as controls (Group I). Twenty-one days after either delivery or termination of treatment all the animals received an intragastric dose of 8 mg DMBA/100 gbw. For the second protocol, 50 day-old virgin rats were also mated (Group V) or were treated with hCG for 21 days (Group VI); the resting period between delivery or termination of treatment was lengthened to 63 days, at which time they received a dose of DMBA. Age-matched controls (Group IV) received DMBA only. Tumourigenesis was evaluated 24 weeks post-carcinogen administration in all the groups. Pregnancy and hCG followed by the 21-day resting period significantly depressed mammary carcinogenesis to 11% and 6% respectively, compared with 63% in control animals. When the resting period was prolonged to 63 days there was also a significant depression in adenocarcinoma incidence to 9% in pregnancy (Group IV) in which it was observed that tumour incidence was also reduced as a consequence of aging at the time of exposure to the carcinogen. These results clearly indicate that hCG is as efficient as pregnancy and significantly reduces mammary carcinogenesis, and that the protective effect of both pregnancy and hCG treatment is long-lasting and both are more efficient than aging in reducing mammary carcinogenesis.


*Br. J. Cancer* (1991), **64**, 481–484 © Macmillan Press Ltd., 1991

### Comparative study of the influence of pregnancy and hormonal treatment on mammary carcinogenesis

I.H. Russo[1,*], M. Koszalka[1] & J. Russo[1]

[1]*Department of Pathology, Michigan Cancer Foundation, 110 East Warren Avenue, Detroit, Michigan 48201, USA.*

**Summary** Since it has been shown that pregnancy protects the mammary gland from chemically induced carcinogenesis, this study was designed with the dual purpose of determining whether treatment of young virgin rats with the placental hormone chorionic gonadotropin (hCG) mimics pregnancy-induced changes in the tumourigenic response of the mammary gland and also whether the effect induced by both pregnancy and hormonal treatments was transitory, or a more permanent one, exerting the same effect when the period of time between delivery or termination of treatment and exposure to the carcinogen is lengthened. Virgin Sprague-Dawley rats were utilised in two experimental protocols. For protocol I, 50 day-old rats were either mated (Group II), or started receiving a daily intraperitoneal injection of 100 IU hCG (Group III) at age 50. Age-matched untreated virgin rats were used as controls (Group I). Twenty-one days after either delivery or termination of treatment all the animals received an intragastric dose of 8 mg DMBA/100 gbw. For the second protocol, 50 day-old virgin rats were also mated (Group V) or were treated with hCG for 21 days (Group VI); the resting period between delivery or termination of treatment was lengthened to 63 days, at which time they received a dose of DMBA. Age-matched controls (Group IV) received DMBA only. Tumourigenesis was evaluated 24 weeks post-carcinogen administration in all the groups. Pregnancy and hCG followed by the 21-day resting period significantly depressed mammary carcinogenesis to 11% and 6% respectively, compared with 63% in control animals. When the resting period was prolonged to 63 days there was also a significant depression in adenocarcinoma incidence to 9% in pregnancy (Group V) and 7% in hCG treated (Group VI) animals respectively, *vs* 18% in control animals (Group IV) in which it was observed that tumour incidence was also reduced as a consequence of aging at the time of exposure to the carcinogen. These results clearly indicate that hCG is as efficient as pregnancy and significantly reduces mammary carcinogenesis, and that the protective effect of both pregnancy and hCG treatment is long-lasting and both are more efficient than aging in reducing mammary carcinogenesis.

Epidemiologic data have shown that a single early full-term pregnancy reduces the lifetime risk of developing breast cancer (MacMahon *et al.*, 1973; Russo & Russo, 1987). Experimental data have shown that pregnancy also protects the mammary gland from chemically induced carcinogenesis (Russo & Russo, 1987; Russo *et al.*, 1982). Therefore, pregnancy can be considered the most physiologic and efficient means of protecting the breast from neoplastic transformation. The goal of protecting the mammary gland by mimicking pregnancy in virgin rats by exogenous treatment could be accomplished by using hormones (Russo *et al.*, 1989*a,b*). We have designed studies for determining whether this protection could be mimicked by treatment of virgin rats with the placental hormone chorionic gonadotropin (hCG), with the objective of aiming at inhibiting tumour initiation at the same level or better than pregnancy. We have already demonstrated that hCG treatment produces a dose-dependent inhibition of mammary carcinomas induced by the chemical carcinogen 7,12-dimethylbenz(a)anthracene (DMBA) (Russo, 1983; Russo *et al.*, 1990*a*). We have also demonstrated that hCG significantly inhibits tumour progression (Russo *et al.*, 1990*a*).

We designed this study with a dual purpose: (1) to compare the efficiency of full-term pregnancy without lactation with that of hCG treatment in inhibiting mammary carcinogenesis, and (2) to determine whether the protective effect of pregnancy or hormonal treatment on mammary carcinogenesis was a permanent one or whether it was ameliorated or lost as the time between the termination of either pregnancy or treatment and the time of exposure to the carcinogen lengthened.

Correspondence: I.H. Russo, *Department of Pathology, Fox Chase Cancer Center, 7701 Burholme Avenue, Philadelphia, PA 19111, USA.
Supported by American Cancer Society grant BC-621 and an Institutional grant from the United Foundation of Greater Detroit.
Received 28 January 1991; and in revised form 29 April 1991.

### Materials and methods

Outbred virgin Sprague-Dawley rats were purchased from Harlan Sprague-Dawley (Indianapolis, IN). The animals were housed four to a cage and were maintained at a temperature of 24 ± 1°C with a 12 h darkness and 12 h light cycle. The animals received water and food ad libitum. When they reached the age of 50 days, they were divided into two experimental protocols (Figure 1).

In protocol 1, 50 day-old animals were divided into Groups I, II and III. Group I consisted of virgin females receiving an intragastric (ig) dose of 7,12-dimethylbenza(a)anthracene (DMBA) (Eastman Organic Chemicals, Rochester, NY) per 100 g of body weight (gbw) when they reached the age of 92 days (Figure 1). Group II animals were mated with 80 day-old Sprague Dawley males. The animals were housed three to a cage, one male and two females. The morning in which a vaginal plug or sperms were found in the vaginal smear was considered day 1 of pregnancy. Pregnant females were separated one to a cage and allowed to deliver their pups. The number of litters delivered was counted and the pups were removed immediately upon delivery. Group III animals consisted of 50 day-old virgin females which were treated with an intraperitoneal injection of 100 IU hCG (Sigma Chemical Co., St Louis, MO) for 21 days. This dose was selected based upon previous results which showed that it induces the maximal protection from mammary carcinogenesis (Russo *et al.*, 1990*a*). Twenty-one days after delivery in animals of Group II or after the last injection for animals of Group III, respectively, the animals received an ig dose of 8 mg of DMBA/100 gbw. DMBA was dissolved in corn oil heated in a water bath at 100°C for 15 min.

For protocol 2, 3 groups of animals, Group IV, controls, Group V, animals mated at age 50, as described for Group II and Group VI, virgin animals treated with 100 IU hCG, as for group III, received an ig dose of 8 mg DMBA/100 gbw 63 days after delivery or hCG treatment (Groups V and VI, respectively), when they were 134 days old (Table I, Figure 1).

All the animals were palpated twice a week for detection of

**Figure 1** Schematic representation of the experimental protocol. Protocol 1 comprises Groups I, II and III. Protocol 2 comprises Groups IV, V and VI. Pregnancy (Preg); resting period (RP); human chorionic gonadotropin (hCG) 100 IU/day for 21 days; 7,12-dimethylbenz(a)anthracene (DMBA).

**Figure 2** Tumour incidence (ordinate): Percentage of animals with tumours (in tens); 7,12-dimethylbenz(a)anthracene (DMBA); Pregnancy (P) + resting period (RP); human chorionic gonadotropin (hCG). ▩ DMBA, ▥ P + 21d-RP + DMBA, ▧ hCG + 21d-RP + DMBA, ■ DMBA, ▦ P + 63d-RP + DMBA, □ hCG + 63d-RP + DMBA.

tumour development. The date of tumour appearance and tumour location were recorded. The final tumourigenic response was evaluated 24 weeks after DMBA administration (Figure 1).

All tumours and the mammary glands were dissected from the skin and processed as described elsewhere (Russo *et al.*, 1989*c*). Sections of tumours were stained with haematoxylin and eosin. Tumours were classified by applying criteria published elsewhere (Russo *et al.*, 1990*b,e* Russo *et al.*, 1989*e*). The proportions of DMBA-induced tumours and DMBA-induced adenocarcinomas were analysed by the chi-square test and Fisher's exact probability two-tail test (Zar, 1984).

### Results

Mammary tumours were observed in all groups of DMBA inoculated animals, although their incidence was significantly depressed by pregnancy, hCG treatment and aging prior to exposure to the carcinogen (Table I). Maximal tumourigenic response was exhibited by control virgin rats (Group I) inoculated with the carcinogen when they were 92 days-old (Table I, Figures 2 and 3).

A significant reduction in tumour incidence was observed in animals in which pregnancy was completed 21 days prior to DMBA treatment (Group II). Age-matched hCG-treated rats (Group III) also developed significantly fewer tumours (Table I, Figures 2 and 3). When tumours were histologically classified it was found that the predominant tumour types were adenocarcinomas and fibroadenomas, both of which were present in both control and hCG treated rats, whereas parous animals (Group II) developed only adenocarcinomas, which were present at the same percentage as in hCG-treated animals (Table I, Figures 2 and 3).

**Figure 3** Adenocarcinoma incidence (ordinate): Percentage of animals developing mammary adenocarcinomas (in tens). Legends as per Figure 2. ▩ DMBA, ▥ P + 21d-RP + DMBA, ▧ hCG + 21d-RP + DMBA, ■ DMBA, ▦ P + 63d-RP + DMBA, □ hCG + 63d-RP + DMBA.

In animals of protocol 2, in which the resting period after delivery or termination of hormonal treatment was prolonged to 63 days, it was observed that both experimental groups exhibited a significant diminution in both tumour and adenocarcinoma incidence in comparison with the age-matched control groups. As it was observed in animals of protocol 1, the total number of tumours in parous animals (Group V) was lower than in hCG treated animals (Group VI). The incidence of adenocarcinomas was significantly reduced by both pregnancy and hCG treatment in com-

**Table I** Effect of hCG[a] on DMBA-induced mammary carcinogenesis

| | | *Tumours* | | | | | *Adenocarcinoma* | | | | |
|---|---|---|---|---|---|---|---|---|---|---|---|
| *Group* | *Treatment* | *Total No. An.* | *No. An.* | *%* | *Total no. tumours* | *No. T/ An.*[b] | *No. An.* | *%* | *Total No. AdCa*[c] | *No. AdCa/An*[d] | *Latency period in days* |
| *Protocol 1* | | | | | | | | | | | |
| I | ---/DMBA[e] | 80 | 49 | 61.3[f] | 101 | 1.26 | 35 | 48.30[g] | 73 | 0.91 | 55–191 |
| II | Pregnancy/DMBA | 18 | 1 | 5.6[f] | 1 | 0.06 | 1 | 5.6[g] | 1 | 0.06 | 91 |
| III | hCG(100 IU)/DMBA | 65 | 19 | 29.2[f] | 27 | 0.41 | 4 | 6.15[g] | 4 | 0.06 | 90–195 |
| *Protocol 2* | | | | | | | | | | | |
| IV | ---/DMBA | 27 | 12 | 44.4[f] | 18 | 0.67 | 5 | 18.52[g] | 5 | 0.18 | 52–153 |
| V | Pregnancy/DMBA | 21 | 3 | 14.28[f] | 4 | 0.19 | 2 | 9.52[g] | 2 | 0.09 | 52–210 |
| VI | hCG(100 IU)/DMBA | 27 | 7 | 25.92[f] | 7 | 0.25 | 2 | 7.40[g] | 2 | 0.07 | 52–210 |

[a]hCG – Human chorionic gonadotropin. [b]Number of tumours. [c]AdCa – Adenocarcinoma. [d]Number of adenocarcinomas per animal per total number of animals at risk. [e]DMBA – 7,12-dimethylbenz(a)anthracene, 8 mg/100 g body weight. [f]Tumour incidence chi-square with d.f. = 3, value 22.4118 and sample size 254 probability is $P = 5.35 \times 10^{-5}$. [g]Carcinoma incidence chi-square with d.f. = 3, value 31.6775 and sample size 254 probability is $P = 6.12 \times 10^{-7}$.

parison with the incidence in age-matched controls (Group IV), but there was no difference in adenocarcinoma incidence between parous and hCG treated animals (Table I, Figures 2 and 3). The protection induced by hCG and pregnancy was similar in animals receiving the carcinogen at different times (Table I, Figures 2 and 3).

Control animals of Group III developed fewer tumours and adenocarcinomas than control animals in Group I, which was due to aging of these animals prior to exposure to the carcinogen (Table I, Figures 2 and 3).

As it has been previously demonstrated (Russo *et al.*, 1982) and confirmed in these results, aging *per se* significantly reduced mammary carcinogenesis, nevertheless pregnancy and hCG treatment were still more effective in reducing both tumour and adenocarcinoma incidence. Although the protection induced by both pregnancy and hCG was longlasting, there appeared to exist a threshold of susceptibility to carcinogenesis which was not modified by pregnancy, hormonal treatment, variations and length of time after completion of pregnancy or treatment or aging, since exposure to the carcinogen always succeeded in inducing at least one carcinoma per group (Table I).

### Discussion

Results presented here demonstrate that both pregnancy and hCG treatment have a similarly protective effect on DMBA-induced mammary carcinogenesis and that this protection is longlasting, since the same reduction in carcinoma incidence is observed when the carcinogen is administered after either 21 or 63 days post-delivery or termination of the hormonal treatment respectively.

Our studies on the pathogenesis and prevention of chemically-induced mammary carcinogenesis have allowed us to conclude that the degree of differentiation of the mammary gland is one of the most important biological characteristics in determining its overall tumourigenic response (Russo & Russo, 1987; Russo *et al.*, 1982, 1990*d*). Maximal susceptibility to DMBA-initiated neoplastic transformation is exhibited by mammary glands of young nulliparous females, which are characterised by the presence of undifferentiated terminal ductal structures or TEBs exhibiting a high rate of cell proliferation. The minimal susceptibility to chemically-induced carcinogenesis exhibited by the mammary gland of parous animals is attributed to its full differentiation (Russo & Russo, 1980, 1987; Russo *et al.*, 1982). Differentiation induced by pregnancy eliminates the targets of the carcinogen, sinced the parous rat mammary gland exhibits a reduction in number or total absence of TEBs and reduction in the proliferative activity of the mammary epithelium, in which the cells shift from a proliferative to a resting or $G_0$ compartment (Russo & Russo, 1980).

Based upon the observed powerful influence of differentiation in inhibiting tumour initiation, our research has been geared to determine what hormone or hormone combinations can mimic pregnancy in stimulating gland differentiation to a degree in which it is protected from chemical carcinogenesis (Russo *et al.*, 1982; 1989*a*,*b*; Russo & Russo, 1987). In comparative studies we have tested the effect of the contraceptive agents norethynodrel-mestranol (Russo *et al.*, 1989*a*) and medroxyprogesterone (MPA) (Russo *et al.*, 1989*b*) a combined oral and an injectable progestagenic contraceptive, respectively, and the placental hormone chorionic gonadotropin (hCG) (Russo *et al.*, 1990*a*). These hormones have been used in identical protocols for treating young virgin rats for 21 days, the length of time of pregnancy, and stopped 21 days prior to carcinogen administration (Russo *et al.*, 1989*a*,*b*, 1990*a*,*c*,*d*). Treatment with various doses of hCG showed a dose-related protective effect (Russo, 1983; Russo *et al.*, 1990*a*), an effect similar to that of norethynodrel-mestranol (Russo *et al.*, 1989*a*), whereas MPA reduced tumour incidence only with the dose clinically used for contraception, but a higher dose resulted in greater tumourigenic response (Russo *et al.*, 1989*b*). Chorionic gonadotropin, like pregnancy, induces full differentiation of the mammary gland, morphologically manifested as a reduction in the number of TEBs, increase in lobular formations and depression of DNA synthesis (Russo *et al.*, 1990*c*).

The use of hormones for inhibiting mammary carcinogenesis has been explored by several authors (Dao *et al.*, 1960; Dao & Sutherland, 1959; Grubbs *et al.*, 1983*a*,*b*, 1986; Huggins *et al.*, 1961, 1962; McCormick & Moon, 1965, 1973; Welsch, 1985). Among the hormones tested for this purpose have been treatment with oestrogen and progesterone at different doses (Grubbs *et al.*, 1983*b*; McCormick *et al.*, 1965), although hormone deprivation through ovariectomy or administration of the anti-oestrogen tamoxifen also inhibits mammary carcinogenesis (Welsch, 1985). In general all these protocols produce protection, although to a different degree. However, none of those treatments can be considered to be a physiologic means for breast cancer prevention, since they modify the reproductive and endocrinologic profile of the animal. The protocols we use for preventing mammary carcinogenesis are unique in the sense that treatments are administered for the same length of pregnancy and they are terminated 21 or more days prior to exposure to the carcinogen, thus testing their effect as permanent modifiers of the mammary gland architecture and cell kinetic properties (Russo *et al.*, 1989*a*,*b*, 1990*a*).

In the present work, the fact that either pregnancy or hCG treatment sufficed to induce a lasting protective effect on the initiation of mammary carcinomas, which was still evident by 63 days post treatment supports the concept that permanent changes have occurred in the mammary gland and are operational in protecting the mammary gland from chemical carcinogenesis, thus allowing to rule out that modifications in circulating hormonal levels (Ciocca *et al.*, 1982), or an immunologic response elicited by the foetus (Sinha *et al.*, 1988) are responsible of the protective effect of pregnancy.

HCG at the doses utilised induces ovulation and maintains the corpus luteum in the ovary (Amsterdam *et al.*, 1975; Rajaniemii *et al.*, 1985; Uilenbrock *et al.*, 1985; Wide *et al.*, 1980), which in turn secretes oestrogens and progesterone, both of which stimulate gland differentiation by mechanisms similar to those operational during pregnancy. The fact that hCG is not tumourigenic, does not induce alterations in body weight or in the weight of endocrine organs such as pituitary gland and adrenal glands, and that after hormone withdrawal the animals return to a normal oestrous cycle (Russo *et al.*, 1990*c*,*d*), indicate that although pregnancy seems to be the most physiologic mechanism for preventing the initiation of mammary carcinogenesis, hCG adequately mimics the effect of pregnancy, making the use of this protocol for cancer prevention an appealing idea that needs further exploration.

### References

AMSTERDAM, A., KOCH, Y., LIEBERMAN, M.E. & LINDNER, H.R. (1975). Distribution of binding sites for human chorionic gonadotropin in the preovulatory follicle of the rats. *J. Cell Biol.*, **67**, 894.

CIOCCA, D.R., PARENTE, A. & RUSSO, J. (1982). Endocrinological milieu and susceptibility of the rat mammary gland to carcinogenesis. *Am. J. Pathol.*, **109**, 47.

DAO, T.L., BOCK, F.G. & GREINER, M.J. (1960). Mammary carcinogenesis by 3-methylcholanthrene II. Inhibitory effect of pregnancy and lactation on tumor induction. *J. Natl Cancer Inst.*, **25**, 991.

DAO, T.L. & SUNDERLAND, H. (1959). Mammary carcinogenesis by 3-methylcholanthrene I. Hormonal aspects in tumor induction and growth. *J. Natl Cancer Inst.*, **23**, 567.

GRUBBS, C.J., HILL, D.L., MCDONOUGH, K.C. & PECKHAM, J.C. (1983*a*). N-Nitroso-N-methylurea-induced mammary carcinogenesis: effect of pregnancy on preneoplastic cells. *J. Natl Cancer Inst.*, **71**, 625.

GRUBBS, C.J., JULIANA, M.M., HILL, D.L. & WHITAKER, L.M. (1986). Suppression of pregnancy by chemically-induced preneoplastic cells of the rat mammary gland. *Anticancer Res.*, **6**, 1395.

GRUBBS, C.J., PECKHAM, J.C. & MCDONOUGH, K.C. (1983*b*). Effect of ovarian hormones on the induction of 1-methyl-1-nitrosourea-induced mammary cancer. *Carcinogenesis*, **4**, 495.

HUGGINS, C., GRAND, L.C. & BRILLANTES, F.P. (1961). Mammary cancer induced by a single feeding of polynuclear hydrocarbons and its suppression. *Nature*, **189**, 204.

HUGGINS, C., MOON, R. & MORII, S. (1962). Extinction of experimental mammary cancer. I. Estradiol-17B and progesterone. *Proc. Natl Acad. Sci. USA*, **48**, 379.

MACMAHON, B., COLE, P. & BROWN, J. (1973). Etiology of breast cancer: a review. *J. Natl Cancer Inst.*, **50**, 20.

MCCORMICK, G.M. & MOON, R.C. (1965). Effect of pregnancy and lactation on growth of mammary tumours induced by 7,12-dimethylbenz(a)anthracene (DMBA). *Br. J. Cancer*, **19**, 160.

MCCORMICK, G.M. & MOON, R.C. (1973). Effect of increasing doses of estrogen and progesterone on mammary carcinogenesis in the rat. *Europ. J. Cancer*, **9**, 483.

RAJANIEMII, H., SOGN, J., HOLMES, P., KALLFELT, B. & JAMSON, P.O. (1985). Fate of receptor-bound human chorionic gonadotrophin in pseudopregnant rat ovaries perfused *in vitro*. *Acta Endocrinol.*, **109**, 115.

RUSSO, I.H., FREDERICK, J. & RUSSO, J. (1989*a*). Hormone prevention of mammary carcinogenesis by norethynodrel-mestranol. *Breast Cancer Res. Treat.*, **14**, 43.

RUSSO, I.H., GIMOTTY, P., DUPUIS, M. & RUSSO, J. (1989*b*). Effect of medroxyprogesterone acetate on the response of the rat mammary gland to carcinogenesis. *Br. J. Cancer*, **59**, 210.

RUSSO, I.H., KOSZALKA, M. & RUSSO, J. (1990*a*). Human chorionic gonadotropin and rat mammary cancer prevention. *J. Natl Cancer Inst.*, **82**, 1286.

RUSSO, I.H., KOSZALKA, M. & RUSSO, J. (1990*c*). Effect of human chorionic gonadotropin on mammary gland differentiation and carcinogenesis. *Carcinogenesis*, **11**, 1849.

RUSSO, I.H., KOSZALKA, M., GIMOTTY, P.A. & RUSSO, J. (1990*d*). Protective effect of chorionic gonadotropin on DMBA-induced mammary carcinogenesis. *Br. J. Cancer*, **62**, 243.

RUSSO, I.H., TEWARI, M. & RUSSO, J. (1989*c*). Morphology and development of the rat mammary gland. In *Integument and Mammary Gland*, Monograph Series on the Pathology of Laboratory Animals, Jones, T.C., Mohr, U. & Hunt, R.D. (eds), p. 233, Springer-Verlag: Berlin.

RUSSO, J. (1983). Basis of cellular autonomy in the susceptibility to carcinogenesis. *Toxicol. Pathol.*, **11**, 149.

RUSSO, J., GUSTERSON, B.A., ROGERS, A.E., RUSSO, I.H., WELLINGS, S.R. & VAN ZWIETEN, M.J. (1990*b*). Comparative study of human and rat mammary tumorigenesis. *Lab. Invest.*, **62**, 244.

RUSSO, J. & RUSSO, I.H. (1980). Influence of differentiation and cell kinetics on the susceptibility of the rat mammary gland to carcinogenesis. *Cancer Res.*, **40**, 2677.

RUSSO, J. & RUSSO, I.H. (1987). Biological and molecular bases of mammary carcinogenesis. *Lab. Invest.*, **57**, 112.

RUSSO, J., RUSSO, I.H., ROGERS, A.E., VAN ZWIETEN, M.J. & GUSTERSON, B.A. (1990*e*). Tumors of the mammary gland. In *Pathology of Tumors of Laboratory Animals*, WHO, Vol. 1, Turusov, V. & Mohr, U. (eds) p. 47. IARC Scientific Publication no. 99 (International Agency for Research on Cancer) World Health Organization, Oxford University Press.

RUSSO, J., RUSSO, I.H., VAN ZWIETEN, M.J., ROGERS, A.E. & GUSTERSON, B.A. (1989*e*). Classification of neoplastic and non-neoplastic lesions of the rat mammary gland. In *Integument and Mammary Gland*. Monograph Series on the Pathology of Laboratory Animals, Jones, T.C., Mohr, U. & Hunt, R.D. (eds), p. 275. Springer-Verlag: Berlin.

RUSSO, J., TAY, L.K. & RUSSO, I.H. (1982). Differentiation of the mammary gland and susceptibility to carcinogenesis. *Breast Cancer Res. Treat.*, **2**, 5.

SINHA, D.K., PAZIK, J.E. & DAO, T.L. (1988). Prevention of mammary carcinogenesis. *Br. J. Cancer*, **57**, 390.

UILENBROCK, J.TH.J., MEIJS-ROELOFS, H.A.M., WOUTERSENE, P.J.A. & 4 others (1985). Changes in ovarian steroidogenesis in prepubertal rats induced to ovulate by low doses of human chorionic gonadotropin. *J. Endocr.*, **107**, 113.

WELSCH, C.W. (1985). Host factors affecting the growth of carcinogen-induced rat mammary carcinomas: a review and tribute to Charles Brenton Huggins. *Cancer Res.*, **45**, 3415.

WIDE, L., HOBSON, B. & WIDE, M. (1980). Chorionic gonadotropin in rodents. In *Chorionic Gonadotropin*, Segal, S.J. (ed.), p. 37. Plenum Press: NY.

ZAR, J.H. (1984). *Biostatistical Analysis*, 2nd ed. Prentice Hall: Englewood Cliffs, N.J.